# Overexpression of TFF3 is involved in prostate carcinogenesis via blocking mitochondria-mediated apoptosis

**DOI:** 10.1038/s12276-018-0137-7

**Published:** 2018-08-23

**Authors:** Jieying Liu, So Youn Kim, Sun Shin, Seung-Hyun Jung, Seon-Hee Yim, Ji Youl Lee, Sug-Hyung Lee, Yeun-Jun Chung

**Affiliations:** 1 Precision Medicine Research Center, Seoul, Korea; 2 Integrated Research Center for Genome Polymorphism, Seoul, Korea; 3 Department of Microbiology, The Catholic University of Korea, Seoul, Korea; 4 Cancer Evolution Research Center, Seoul, Korea; 5 Department of Urology, The Catholic University of Korea, Seoul, Korea; 6 Department of Pathology, College of Medicine, The Catholic University of Korea, Seoul, Korea

## Abstract

The overexpression of trefoil factor family 3 (TFF3) is observed in a variety of cancers, including prostate cancer (PCa), and its potential role in carcinogenesis, such as activating the PI3K/AKT pathway, is suggested. However, its role and its related mechanisms in prostate tumorigenesis remain unknown. To elucidate the role of TFF3 overexpression in PCa, we silenced TFF3 in two PCa cell lines that overexpressed TFF3 and explored the molecular mechanism behind its antiapoptotic role. We also examined TFF3 expression in 108 Korean PCa specimens and 106 normal prostate tissues by immunohistochemistry (IHC) analysis. The mean TFF3 IHC score in the tumor tissues was significantly higher than that in the normal tissues (4.702 vs. 0.311, *P* = 2.52 × 10^-24^). TFF3-silenced cells showed suppressed tumor cell growth and migration. TFF3 silencing decreased BCL2 and increased BAX expression. The translocation of BAX to the mitochondria was also confirmed. After TFF3 silencing, the expression of the mitochondrial proapoptotic proteins, cytochrome C and Smac/DIABLO, was elevated, and these proteins were released from the mitochondria to the cytosol. Downstream mediators of mitochondrial apoptosis, including cleaved caspase-3, caspase-9, and PARP, were also elevated. Accordingly, the proportion of apoptotic cells was significantly higher among TFF3-silenced cells. There was no difference in extrinsic apoptosis-related molecules after TFF3 silencing. All the results support that TFF3 silencing induces the downstream signaling pathway of mitochondria-mediated apoptosis. This study provides a better understanding of the mechanism of prostate tumorigenesis, suggesting TFF3 as a potential biomarker and therapeutic target of PCa.

## Prostate cancer: Promising protein biomarker

A protein found in high levels in prostate cancer tissues could provide a biomarker and therapeutic target for the disease. Prostate cancer (PCa) is a common cause of cancer-related death, yet existing biomarkers cannot reliably predict PCa risk. The search for a novel biomarker led Yeun-Jun Chung at the Catholic University of Korea in Seoul, South Korea, and co-workers to examine the role of the trefoil factor family 3 (TFF3) protein. TFF3 overexpression is implicated in tumor invasion and in limiting tumor cell death in many cancers, including PCa. Chung’s team analyzed TFF3 levels in 108 cases of PCa and found they were significantly higher than in normal prostate tissues. Blocking the overexpression of TFF3 in two PCa cell lines limited tumor cell growth and migration, and increased cancer cell death.

## Introduction

Prostate cancer (PCa) is the second most common malignancy in men worldwide and is the second most common cause of cancer-related death in men in the US^1,2^. Currently, prostate-specific antigen (PSA) in serum is the most commonly used screening marker for PCa. However, PSA levels cannot reliably predict the risk of PCa^3,4^, and there is no global consensus of a single cutoff value of serum PSA for determining PCa^5,6^. Several genetic alterations are observed to occur frequently in PCa, and these alterations are potential biomarkers for PCa. For example, the *TMPRSS2–ERG* gene fusion occurs in 40–50% of primary PCa tissues^7^, and over 90% of PCa specimens show overexpression of PCA3^8^. However, their clinical utility is still controversial^9^. The allelic loss of 8p12–21 is reported to commonly occur in PCa (>90%) and high-grade prostatic intraepithelial neoplasia (>60%)^10^. The loss of *NKX3–1*, located at 8p12–21, is also reported to be associated with cancer progression and the poor prognosis of PCa^11,12^. Recent next generation sequencing analyses of PCa reveal recurrent somatic mutations, such as *SPOP*, *MED12*, and *FOXA1*^13–16^. However, since PCa has variable biological backgrounds, more reliable biomarkers are required to understand its tumorigenesis mechanism and realize precision medicine for PCa.

Trefoil factor family 3 (TFF3) belongs to the trefoil factor family, which includes two other members (TFF1 and TFF2). TFF3 is a secreted peptide that is predominantly expressed in the mucous epithelia of the gastrointestinal tract^17^. TFF3 and other TFF members are known to be involved in the protection of the gastrointestinal tract against mucosal injury and subsequent repair^18^. In addition to mucosal restitution, TFFs are known to be involved in the migration/invasion of tumor cells, antiapoptosis signaling and the prevention of anoikis in epithelial cells^18–20^, which are the key features of cancer progression. Alterations in TFF3 expression are observed in diverse cancers, such as breast^21,22^, gastric^23^, pancreatic^24^, colorectal^25^, and prostate cancers^26^. Specifically, TFF3 is reported to be commonly overexpressed in PCa.

Garraway et al. and Faith et al. consistently reported that TFF3 was significantly overexpressed in PCa tissues compared with normal prostate tissues (42% vs. 10 and 47% vs. 18.8%, respectively), suggesting that TFF3 is a useful biomarker for PCa^26,27^. Regarding its oncogenic roles, Perera et al. reported that the overexpression of TFF3 significantly increased cell proliferation, anchorage-independent growth, 3-dimensional colony formation, wound healing, cell migration, and radio-resistance^28^. However, the clinicopathologic features and oncogenic mechanisms associated with TFF3 overexpression in PCa are not clear^26,27^, suggesting the necessity of further investigation into its biological roles and underlying mechanisms.

In this study, we aimed to elucidate the roles of TFF3 overexpression in prostate tumorigenesis by knocking down the overexpressed TFF3. We also explored the molecular mechanisms behind its tumorigenic roles and determined that TFF3 is involved in prostate carcinogenesis via blocking the mitochondria-mediated apoptosis pathway.

## Materials and methods

### Cell lines

The LNCap.FGC (hereinafter called LNCap), PC-3, and WPMY-1 (prostate stromal cell line) cells were purchased from the American Type Culture Collection (ATCC, Manassas, VA). They were maintained in RPMI 1640 with 10% FBS (LNCap and PC-3) or DMEM with 5% FBS (WPMY-1).

### Transfection of TFF3 siRNAs

Three different TFF3-specific siRNAs (siTFF3-1, siTFF3-2, and siTFF3-3) were purchased from Invitrogen (Carlsbad, CA). Their sequences are available in Supplementary Table S1. To estimate the sequence-specific effectiveness of the TFF3-specific siRNAs, we also used a negative control siRNA (siCtrl) (Invitrogen) that has no significant homology with any known sequences in the human genome. Since siTFF3-3 showed the best performance of the three constructs (data not shown), we used siTFF3-3 for all the downstream experiments (hereinafter called siTFF3). siTFF3 was transfected into the cells at a final concentration of 50 nM using Lipofectamine® 2000 transfection reagent (Invitrogen). The cells were seeded in growth medium at a density of 40–50% one day before transfection. The cells were harvested at different time points for the following tests.

### TFF3-specific qRT-PCR

Total RNA was isolated from the cells with TRIzol reagent (Invitrogen). Total RNA (5 μg) was reverse transcribed using oligo dT primers and SuperScript® III reverse transcriptase (Invitrogen). Quantitative real-time reverse transcription-PCR (qRT-PCR) was performed with the ViiA™ 7 Real-Time PCR System using THUNDERBIRD™ SYBR® qPCR Mix (Toyobo, Osaka, Japan) and the TFF3-specific primer set: TFF3-F, 5′-CCC TGC AGG AAG CAG AAT-3′ and TFF3-R, 5′-GGG AGC AAA GGG ACA GAA A-3′. GAPDH was used for normalization.

### Western blot analysis

Seventy-two hours following transfection with TFF3-siRNA, the cells were harvested and lysed in RIPA cell lysis buffer with EDTA and a protease inhibitor (GenDEPOT, Houston, TX). The resulting supernatant was collected as total cellular protein and electrophoresed on 12–15% SDS-polyacrylamide gels. The separated proteins were transferred to Immobilon-P polyvinylidene difluoride membranes (Millipore, Billerica, MA) using a BioRad Mini-PROTEAN® Tetra Cell (BioRad, Hercules, CA). The membranes were blocked in 5% skim milk and then incubated overnight at 4 ℃ with antibodies, including a rabbit polyclonal anti-TFF3 antibody (Santa Cruz, Dallas, TX), a mouse monoclonal anti-TFF3 antibody (Santa Cruz), a rabbit monoclonal anti-cleaved caspase-3 antibody (Cell Signaling Technology, Danvers, MA), a rabbit polyclonal anti-caspase-9 antibody (Cell Signaling Technology), a rabbit polyclonal anti-PARP-1 antibody (Santa Cruz), a mouse monoclonal anti-BCL2 antibody (Santa Cruz), a rabbit monoclonal anti-BAX antibody (Abcam, Cambridge, UK), a mouse monoclonal anti-COX IV antibody (Abcam), and a mouse monoclonal anti-β-actin antibody (Sigma, St. Louis, MO). The membranes were washed and incubated with HRP-conjugated goat anti-rabbit IgG or goat anti-mouse IgG antibody (Santa Cruz) for 1 h at room temperature. The blots were detected with SuperSignal™ West Pico Chemiluminescent Substrate (Thermo Fisher Scientific, Waltham, MA) and SuperSignal West Femto Chemiluminescent Substrate (Thermo Fisher Scientific). Blot images were obtained using the Luminescent Image Analysis System (ImageQuant LAS 4000, Fujifilm GE Healthcare Bio-Sciences, Pittsburgh, PA).

### Subcellular fractionation

The cytosol and mitochondrial fractions were prepared using the Mitochondria/Cytosol Fractionation Kit (BioVision, Milpitas, CA) according to the manufacturer’s method.

### Immunocytochemistry

The cells were seeded onto two-well chamber slides and transfected for 72 h with TFF3-siRNA and siCtrl. After blocking with 1% BSA in TBS, the cells were incubated overnight at 4 ℃ with primary antibodies, including a rabbit polyclonal anti-TFF3 antibody (Santa Cruz), a rabbit monoclonal anti-cleaved caspase-3 antibody (Cell Signaling Technology), a mouse monoclonal anti-cytochrome c antibody (Santa Cruz) and a goat polyclonal anti-Smac antibody (Santa Cruz). The slides were rinsed with TBS and then incubated for 1 h with anti-rabbit IgG-FITC (Sigma), anti-mouse IgG-FITC (Santa Cruz) and anti-goat IgG-FITC (Santa Cruz). Then, the cell nuclei were stained with Hoechst 33342 (Life Technologies, Carlsbad, CA), and the mitochondria were stained with the Mito-ID Red detection kit (Enzo Life Sciences, Farmingdale, NY). The slides were analyzed by fluorescence microscope (Axio Imager M1, Carl Zeiss, Oberkochen, Germany). Three fields were randomly selected from three independent experiments.

### BrdU cell proliferation assay

Cell proliferation was investigated by a Cell Proliferation ELISA, BrdU kit (Roche, Mannheim, Germany). The cells were seeded in 96-well plates (8 × 10^3^ to 10^4^ cells per well) in triplicate and then transfected with TFF3-siRNA. The cells were incubated with BrdU for 2 h at 37 ℃ at different time points after transfection (24, 48, 72, and 96 h). The level of BrdU incorporation was quantified by anti-BrdU antibody treatment, and the optical density was measured at 370 nm with a Multilabel Counter System (Perkin Elmer, Waltham).

### Wound healing assay

Seventy-two hours following transfection with TFF3-siRNA, the cells were wounded with a 200 μl pipette tip. Images were taken with a phase-contrast microscope at different time points (0, 24, 48, 72, and 96 h). The distance from the wound edge to the original wound site was measured using ImageJ software^29^.

### Caspase-3 activity assay

Caspase-3 activity was determined by the caspase-3 substrate (DEVD-ρNA) following the instructions in the Caspase-3/CPP32 Colorimetric Assay kit (BioVision, Milpitas, CA). Seventy-two hours after transfection, the cells were harvested and lysed with cell lysis buffer. The protein concentration was determined by the Bradford assay. Fifty microliters of lysate containing 100 μg of protein was mixed with 50 μl of 1× reaction buffer (containing 10 mM DTT). Five microliters of DEVD-pNA substrate was added into the mixture, and it was incubated at 37 ℃ overnight. The absorbance was measured with a Multilabel Counter System (Perkin Elmer).

### Apoptosis assay by flow cytometric analysis

After transfection with siTFF3 and siCtrl (48–72 h), the apoptotic cells were analyzed using the ApoScan TM Annexin V-FITC apoptosis detection kit (BioBud, Sungnam, Korea). Briefly, 10^6^ cells were washed once with cold PBS and resuspended in 0.5 ml of 1× binding buffer. A total of 1.25 μl of Annexin V-FITC was added to the cell suspension, which was incubated for 15 min in the dark, followed by the addition of 10 μl of propidium iodide for 10 min before flow cytometric analysis.

### Immunohistochemistry of PCa tissue microarray

We used a PCa tissue microarray (TMA) developed by the Korea Prostate Bank (Seoul, Korea). This TMA contains 108 cancer tissues and 106 matched normal prostate tissues. In two PCa cases, matched normal tissue was not available. Approval for this study was obtained from the Institutional Review Board of the Catholic University of Korea, College of Medicine. All the cores from the tumor tissue blocks were verified to contain tumor cells by histological examination. The TMA blocks were cut in 4 μm sections and were used for the immunohistochemistry (IHC) analysis. Endogenous peroxidase was blocked with 0.1% H_2_O_2_. The section slides were subjected to microwave antigen retrieval (10 mM citrate buffer, pH 6.0). Then, the slides were incubated with the primary antibody (anti-TFF3 antibody, Santa Cruz, 1:50), the biotinylated secondary antibody, and streptavidin-horseradish peroxidase. A diaminobenzidine solution was used as the chromogen. The slides were counterstained in a hematoxylin solution. The intensity of the TFF3-specific staining was semiquantitatively scored on a 4-point scale as follows: 0 (negative); 1 (weak); 2 (moderate); and 3 (intense). The staining extent was graded according to the percentage of positive staining as follows: 0 (1–5%); 1 (6–19%); 2 (20–49%); and 3 (<50%). Subsequently, the IHC score (IS) was defined from 0 to 9 by multiplying the staining intensity by the staining extent. The IS was used to generate one negative group (IS 1–3) and two positive groups (1+ for IS 4–5 and 2+ for IS 6–9).

### Statistical analysis

A two-tailed unpaired Student’s *t*-test was performed to compare data between groups using SPSS (version 18). *P* < 0.05 was considered statistically significant.

## Results

### Expression of TFF3 in primary human prostate cancers

**Fig. 1:**
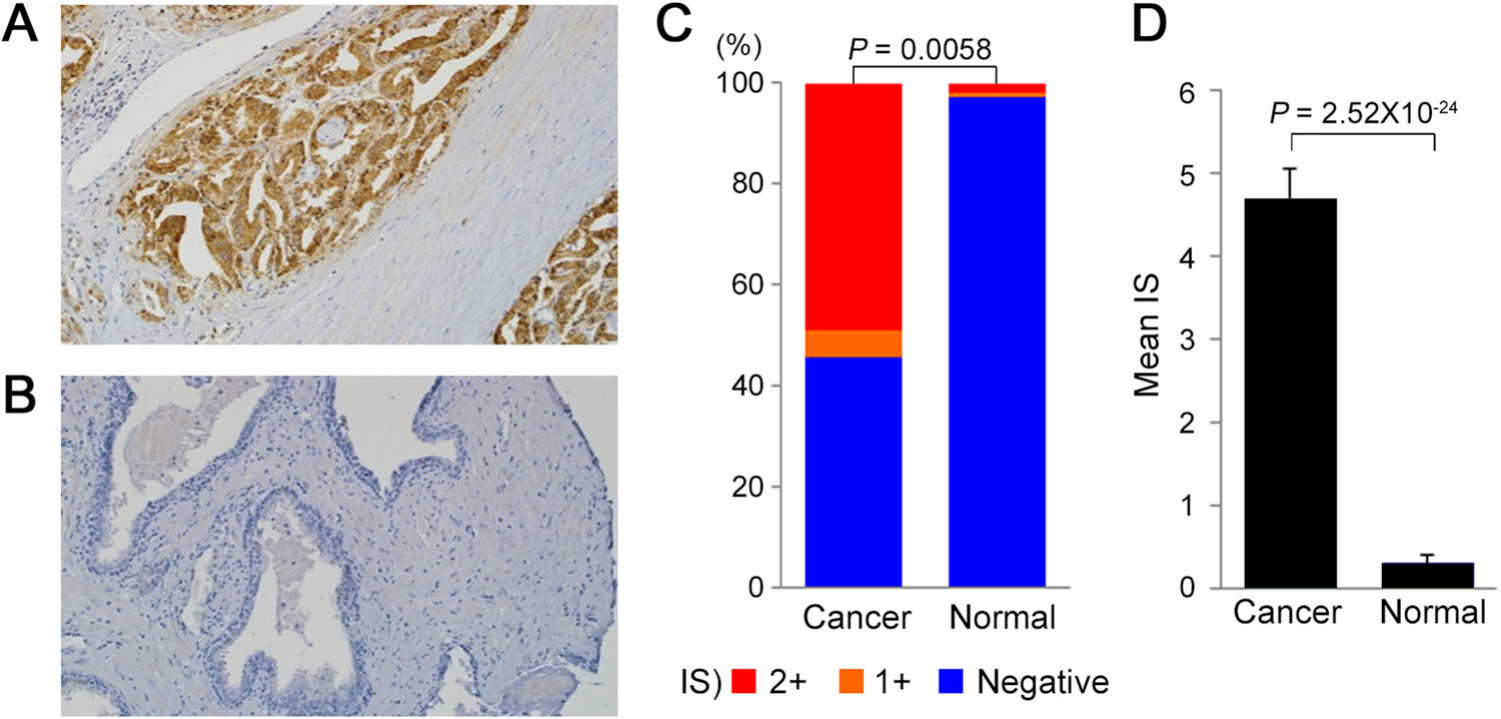
TFF3 protein expression by immunohistochemistry. **a** An example of TFF3 overexpression in PCa cells (100×). **b** An example of TFF3 expression in normal prostate tissues (100×). **c** A proportion of TFF3 IHC score [IS] positives and negatives in PCa and normal prostate tissues. The IS positive (IS 1+ and 2+) proportion of TFF3 in PCa tissues was significantly higher than that in the normal tissues (*P* = 0.0058). **d** The mean TFF3 IS in the PCa and normal tissues. The mean TFF3 IS in the tumor tissues was significantly higher than that in the normal ones (4.702 vs. 0.311, *P* = 2.52 × 10^−24^)

TFF3 protein expression was examined by immunohistochemistry (IHC) using a tissue microarray (TMA) containing 108 cancer tissues and 106 matched normal prostate tissues. The mean age at prostatectomy was 63 years (range, 43–77), and the Gleason scores at biopsy were ≥7 for 90.6% (96/106) of the patients (Supplementary Table S2). Exemplary results of the TFF3 IHC in PCa and normal prostate tissues are illustrated in Fig. 1a, b. The grading of the IHC score (IS) and the assessment of the TFF3 status were performed as described in the Materials and Methods. The TFF3-positive proportion in the PCa tissues was significantly higher than that in the normal prostate tissues (54.6% vs. 2.8%, *P* = 0.0058) (Fig. 1c, Supplementary Table S3). The proportion of strong positives (IS > 2+) was also significantly higher in the PCa tissues than in the normal tissues (49.1% vs. 1.9%, *P* = 0.0062). Moreover, the mean TFF3 IS in the tumor tissues was significantly higher than that in the normal tissues (4.702 vs. 0.311, *P* = 2.52 × 10^-24^) (Fig. 1d). Even the TFF3-negative tumor tissues showed a relatively higher IS (44.9% for IS 1–3 and 55.1% for IS 0) than did the normal tissues (13.6% for IS 1–3 and 86.4% for IS 0). The details of the IHC scores in the PCa and normal prostate tissues are available in Supplementary Table S3.

### The effects of TFF3 silencing on the proliferation and migration of prostate cancer cells

**Fig. 2:**
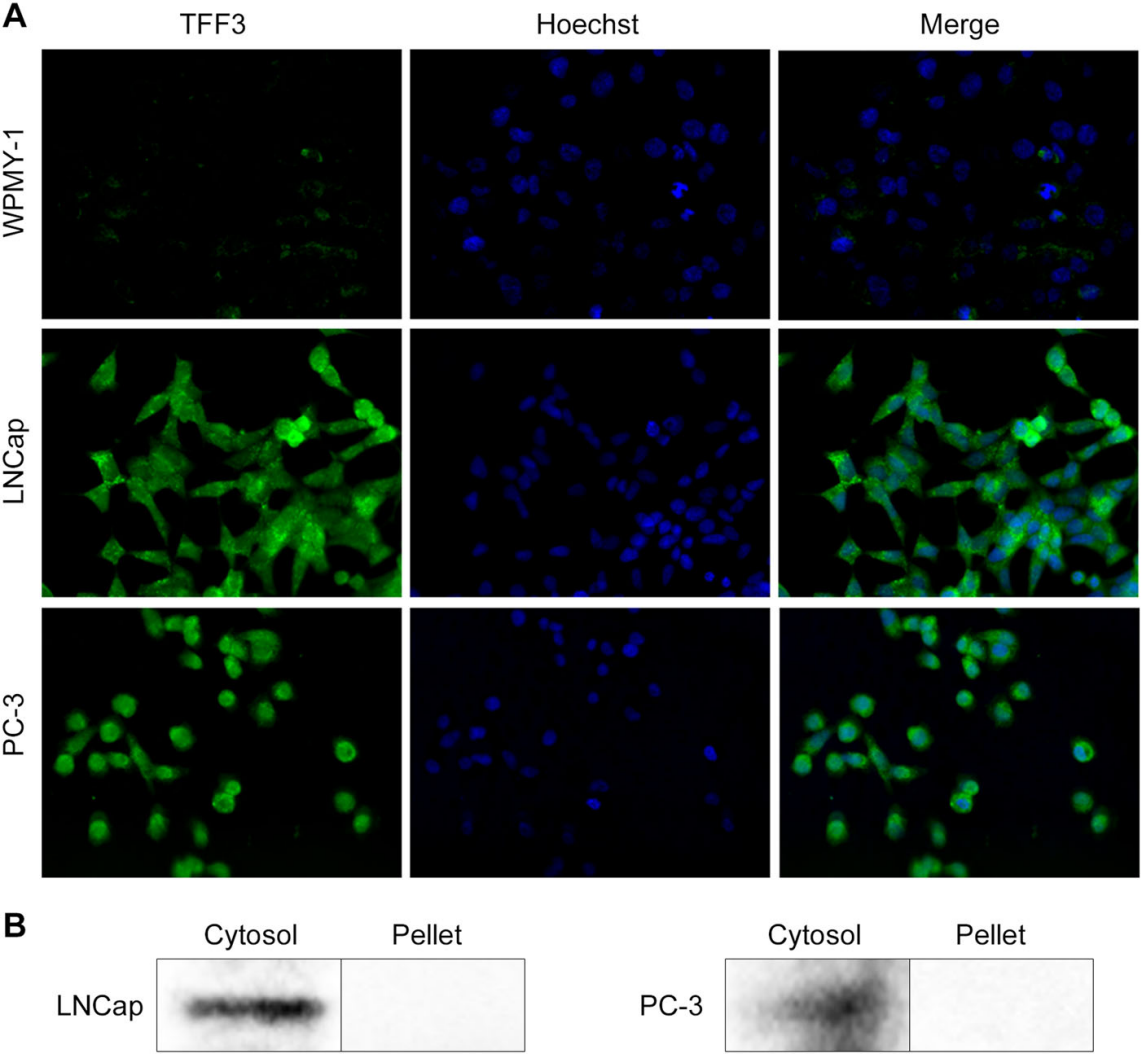
Cytosolic localization of TFF3. **a** TFF3 expression and subcellular localization in PCa cell lines (LNCap and PC-3) and a normal prostate cell line (WPMY-1) identified by immunocytochemistry. TFF3 (green fluorescence); Nucleus (Hoechst 33342, blue). **b** The cytosolic expression of TFF3 was confirmed by western blot analysis of subcellular fractions in LNCap and PC-3 cells

We examined the mRNA expression levels of TFF3 in two prostate cancer cell lines (PC-3 and LNCap) and one normal prostate cell line (WPMY-1) by qRT-PCR. Both LNCap and PC-3 cells showed 7800 and 3500-times higher levels of TFF3 expression than the WPMY-1 cells, respectively (Supplementary Figure S1). When we examined TFF3 expression by immunocytochemistry analysis, TFF3 expression in the PC-3 and LNCap cells was much stronger than that in WPMY-1 cells, which is consistent with the qRT-PCR and IHC results, and TFF3 was mainly localized in the cytoplasm (Fig. 2a). Consistent with the immunocytochemistry result, TFF3 was predominantly detected in the cytosolic fractions of both cell lines (Fig. 2b).

**Fig. 3:**
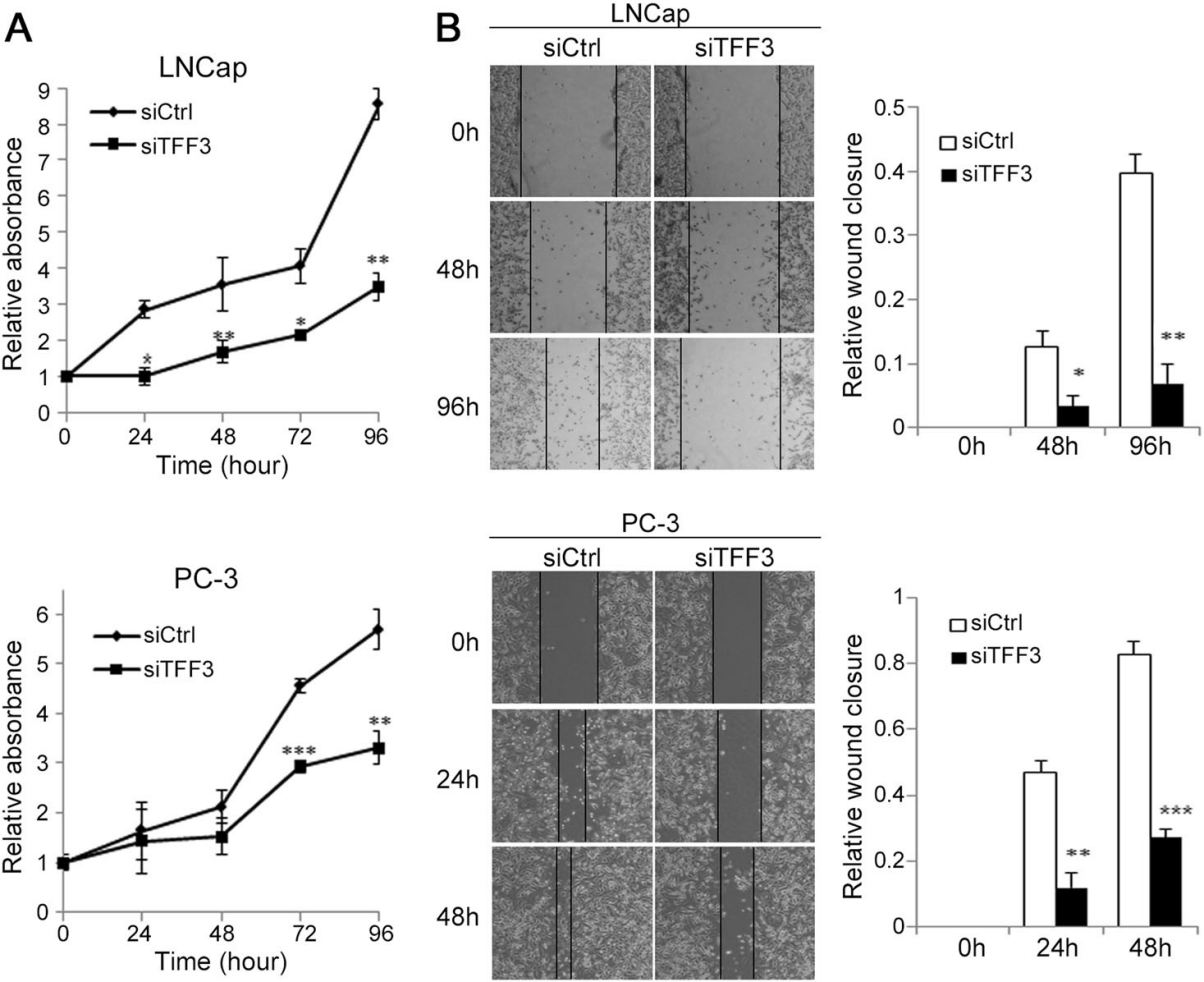
Effect of silencing overexpressed TFF3 on PCa cell growth and motility. **a** The effect of TFF3 silencing on the proliferation of LNCap and PC-3 cells was measured by a BrdU incorporation assay at the indicated times (0, 24, 48, 72, and 96 h) after transfection with siTFF3 or siCtrl. The proliferation of both TFF3-silenced cell lines was significantly attenuated compared with that of the non-silenced cells. The relative absorbance value is the mean ± SEM of the results from three independent experiments. *P < 0.05; **P < 0.01; ***P < 0.001. **b** The effect of TFF3 silencing on cell migration. The images show cell migration monitored by a scratch wound healing assay at different time points (0, 24, 48, and 96 h) after transfection. The bar charts at the right side of the images represent the relative wound closure. The relative wound closure was calculated using the following formula: wounded area invaded by cells/wounded area at 0 h. The migration of TFF3-silenced cells was significantly diminished compared to that of the non-silenced cells. The relative wound closure value is the mean ± SEM of the results from three independent experiments. *P < 0.05; **P < 0.01; ***P < 0.001

To investigate the role of TFF3 overexpression in prostate carcinogenesis, we examined the proliferation of PCa cells after the knockdown of overexpressed TFF3 by transfecting the PCa cell lines with TFF3-specific siRNA (siTFF3). The growth of TFF3-silenced PC-3 and LNCap cells was significantly repressed compared with that of non-silenced cells treated with control siRNA (siCtrl) (Fig. 3a). To explore whether TFF3 affects cell migration, we examined cell motility using the scratch wound healing migration assay. Similar to the proliferation assay results, TFF3-silenced cells showed significantly diminished migration and motility (Fig. 3b). TFF3-silenced LNCap cells showed decreased migration by 3.8-fold at 48 h and 5.8-fold at 96 h relative to control cells. Likewise, TFF3-silenced PC-3 cells manifested reduced migration by 3.9-fold at 24 h and 3.1-fold at 48 h relative to control cells. Decreased migration was also observed at earlier time points (6 and 12 h) but was less prominent (Supplementary Figure S2).

### Molecular mechanism behind the oncogenic role of TFF3

**Fig. 4:**
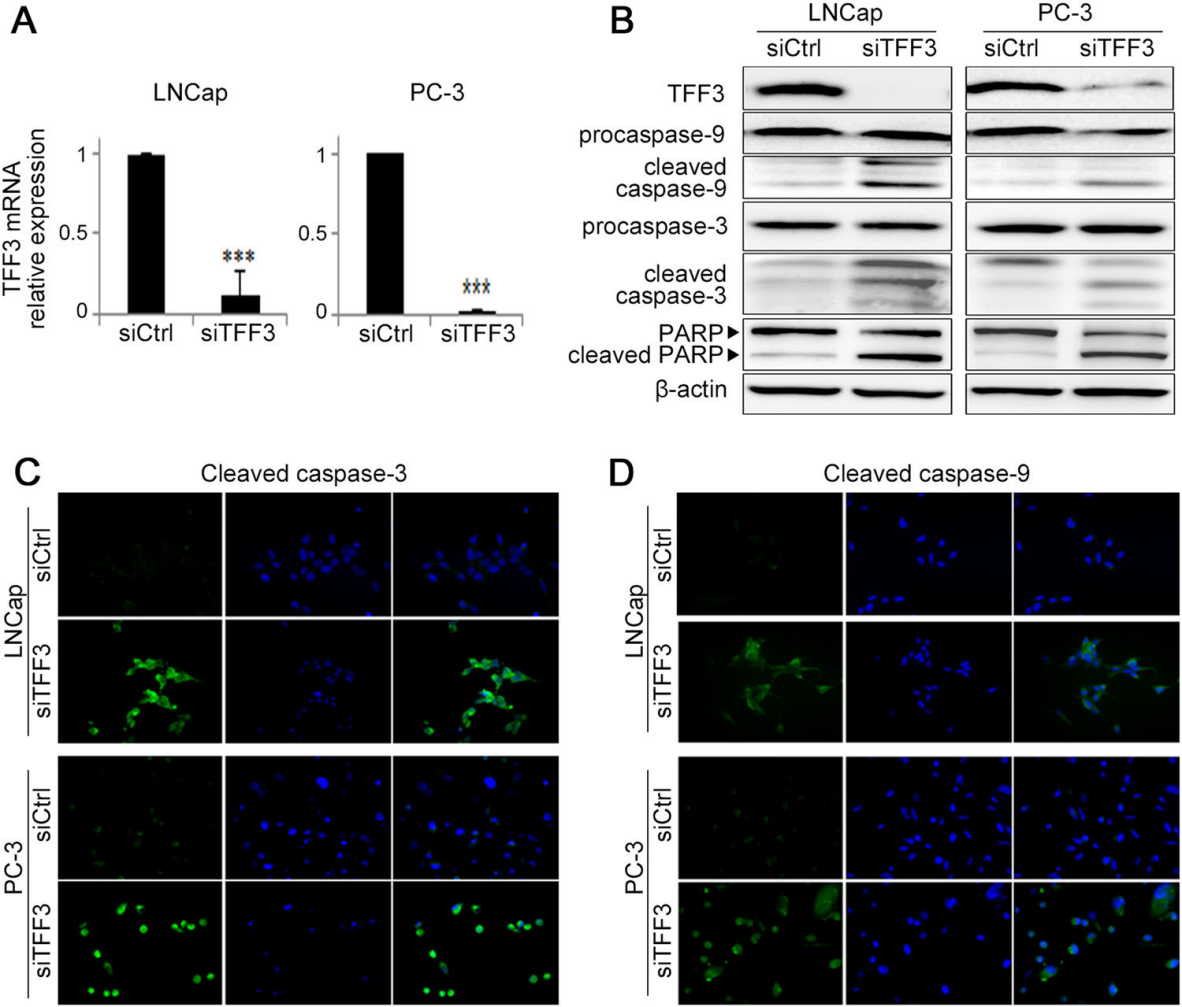
Effect of silencing overexpressed TFF3 on the expression of apoptosis-related proteins. **a** TFF3 expression after TFF3 silencing. The relative levels of TFF3 mRNA expression at 72 h after siRNA treatment were measured by real-time RT-PCR. The mRNA expression of TFF3 was downregulated by over 90% (***P < 0.001) after silencing TFF3 in both cells. **b** The western blot analysis shows that cleaved caspase-3 and caspase-9 are increased but that procaspase-3 and procaspase-9 levels did not change after TFF3 silencing. The level of cleaved PARP, another hallmark of programmed cell death, also increased. **c**, **d** The immunocytochemistry shows elevated cleaved caspase-3 and caspase-9 (green) levels in TFF3-silenced cells compared with non-silenced cells (400×). Nuclei (blue: Hoechst 33342)

After siTFF3 transfection, TFF3 expression (at both the mRNA and protein expression levels) was significantly downregulated by over 90% compared with that in siCtrl-treated cells (Fig. 4a, b). When we examined whether TFF3-silencing inhibits the phosphorylation of AKT-1 in PCa cells, the pAKT-1 level was decreased by siTFF3 in a dose-dependent manner (Supplementary Figure S3). To understand the AKT-related downstream molecular mechanisms, we examined changes in the apoptotic signaling molecules caspase-3, caspase-9, and PARP in response to TFF3 repression. As shown in Fig. 4b–d, the levels of cleaved caspase-3, cleaved caspase-9 and cleaved PARP were elevated in siTFF3-transfected cells compared with control cells in both the western blot and immunocytochemistry analyses.

**Fig. 5:**
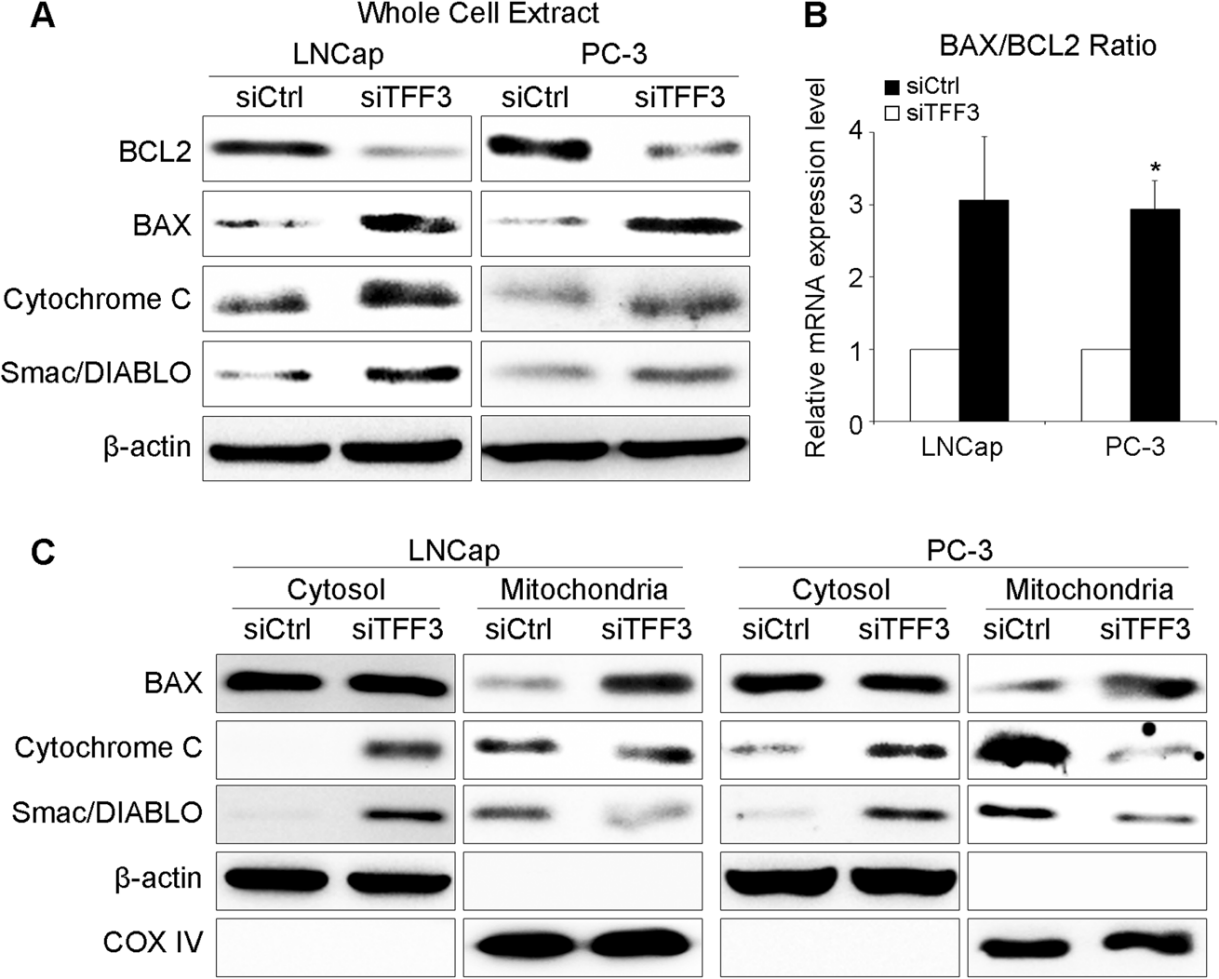
Expression and localization of mitochondrial apoptosis-related regulators after TFF3 silencing. **a** The total protein levels of BCL2 and the proapoptotic factors cytochrome C, Smac/DIABLO and BAX. Expression of the antiapoptotic protein BCL2 was suppressed, and proapoptotic protein expression was increased in TFF3-silenced PCa cells. **b** The relative mRNA expression levels of BCL2 and BAX. The BAX/BCL2 ratio was increased in TFF3-silenced cells. **c** Localization of cytochrome C, Smac/DIABLO and BAX. In the mitochondrial fractions, the BAX level was relatively higher in TFF3-silenced cells than in control cells, suggesting that the translocation of BAX to the mitochondria was increased. In the cytosolic fractions, cytochrome C and Smac/DIABLO expression was relatively higher in TFF3-silenced cells than in control cells, but the inverse was true in the mitochondrial fractions, suggesting that these proteins were released from the mitochondria into the cytosol. Beta-actin and cytochrome oxidase IV (COX IV) were used as loading controls for the cytosolic and mitochondrial fractions, respectively

TFF3-silenced PCa cells showed decreased BCL2 and increased BAX (Fig. 5a). The BAX/BCL2 ratio at the mRNA level was increased in TFF3-silenced cells (Fig. 5b). When we compared the BAX level in the mitochondria and cytosol by subcellular fractionation analysis, there was no difference in cytosolic BAX levels between TFF3-silenced and control cells, but in the mitochondrial fraction, the BAX level was profoundly higher in TFF3-silenced cells (Fig. 5c), suggesting the translocation of BAX to the mitochondria. We next observed mitochondrial proapoptotic proteins (cytochrome C and Smac/DIABLO) in TFF3-silenced cells. In the whole cell extract, cytochrome C and Smac/DIABLO were elevated (Fig. 5a). Through the subcellular fractionation analysis, we confirmed that cytochrome C and Smac/DIABLO were released from the mitochondria to the cytosol in TFF3-silenced cells (Fig. 5c). These phenomena were consistently observed in the immunocytochemistry analyses (Supplementary Figure S4).

To verify whether the apoptosis after siTFF3 treatment was related to inducing extrinsic apoptosis, we examined the expression of three death receptors (TNFR1, FAS and DR4), and none of them showed a significant difference between TFF3-silenced and control cells (Supplementary Figure S5A). Additionally, the expression levels of FADD, TNF-alpha and cleaved caspase-8 were not different after TFF3 silencing (Supplementary Figure S5A, B). In the FADD immunoprecipitation, no FADD-dependent recruitment of procaspase-8 was observed in TFF3-silenced cells (Supplementary Figure S5C).

**Fig. 6:**
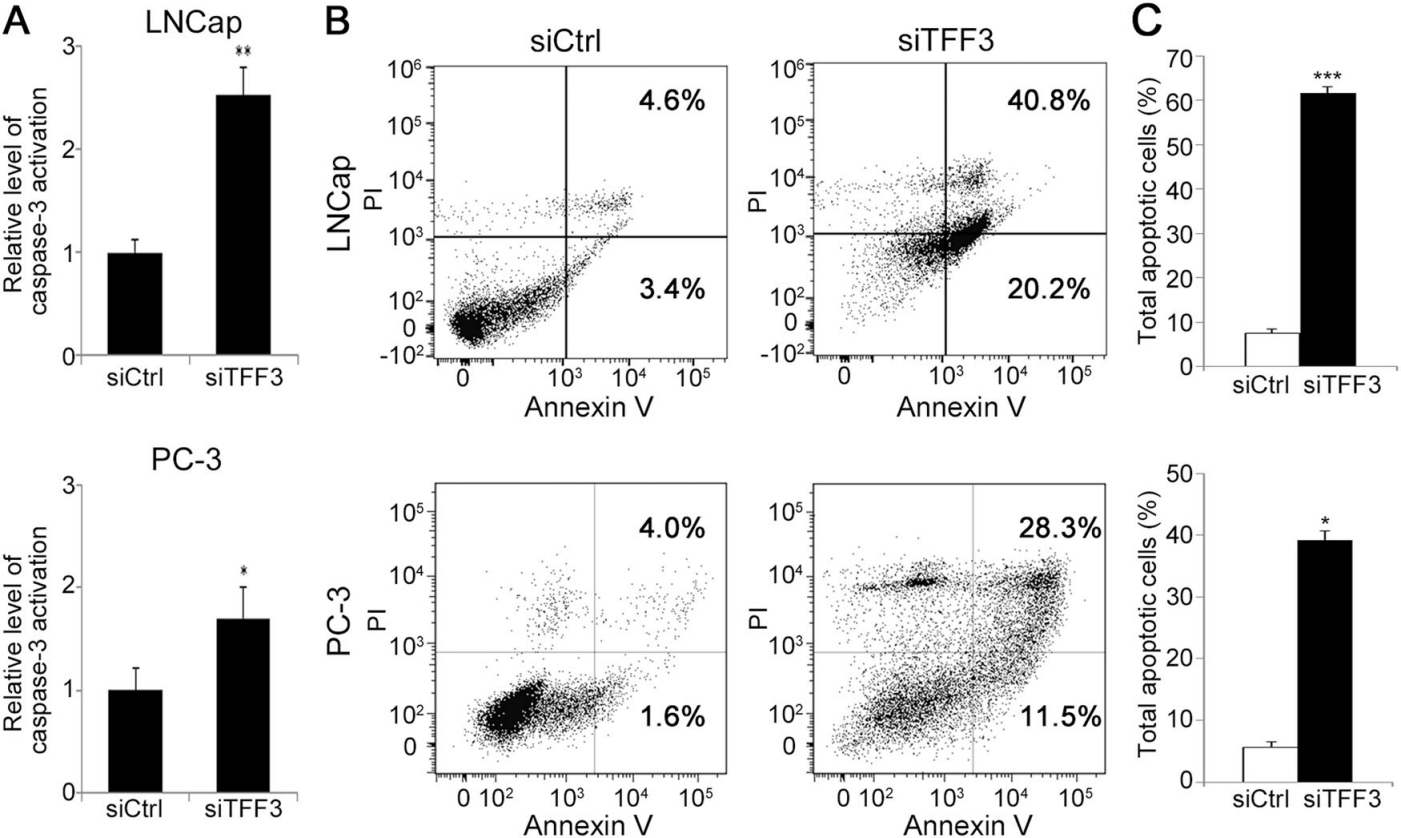
Effect of TFF3 silencing on the expression of activated caspase-3 and Annexin V. **a** Active caspase-3 levels were significantly elevated in both siTFF3-treated cells compared with siCtrl-treated cells. *P < 0.05; **P < 0.01 **b** Representative plot of the FACS analysis. The proportion of Annexin V-positive cells, as measured by the FACS analysis, was higher among siTFF3-transfected cells than among siCtrl-treated cells. **c** The bar chart illustrates the percentage of total apoptotic cells among those transfected with siTFF3 (black bar) or siCtrl (open bar). The percentage of total apoptotic cells is the mean ± SEM of the results from three independent experiments. *P < 0.05; ***P < 0.001

We then measured the activity of caspase-3, an executioner caspase for apoptosis. Consistent with the western blotting result for the apoptosis pathway molecules, active caspase-3 levels were significantly elevated in both of the siTFF3-treated cells compared with the control cells (Fig. 6a, *P* < 0.01 in LNCap; *P* < 0.05 in PC-3). To further verify whether that cell death was induced by TFF3 silencing, we observed the expression of Annexin V by FACS analysis. As shown in Fig. 6b, c, the proportion of Annexin V-positive cells among siTFF3-transfected cells was higher than that among the control cells (61.0% and 39.8% in siTFF3-transfected LNCap and PC-3 cells vs. 7.9% and 5.6% in siCtrl-treated cells).

Regarding the diminished migration after TFF3 silencing, we checked the expression of migration-related molecules, including MMP2, MMP9, CXCR1, CXCR4, CXCL12, and IL8, by qRT-PCR. The expression levels of MMP2 and MMP9 were not different, but the expression of CXCR1, CXCR4, CXCL12 and IL8 was downregulated in TFF3-silenced cells (Supplementary Figure S6).

## Discussion

Although several biomarkers, such as PSA and TMPRSS2–ERG fusion, have been identified for PCa, the molecular mechanisms behind PCa carcinogenesis are still largely unknown. In our previous study exploring the intratumoral genetic heterogeneity of PCa^16^, TFF3 was overexpressed across multiple cases of PCa, suggesting that the overexpression of TFF3 may be an early and common event in PCa tumorigenesis. The aim of this study was three-fold. First, we aimed to identify the profiles of TFF3 protein expression in Korean PCa patients. Second, we aimed to elucidate the roles of TFF3 overexpression in prostate tumorigenesis. Third, we attempted to find the molecular mechanisms behind its antiapoptotic role. By an IHC analysis of 108 Korean PCa cases, we found that TFF3 was significantly overexpressed in PCa, which was consistent with the results of previous reports^26–28,30,31^. After observing the effects of TFF3 silencing on prostate tumorigenesis in PCa cell lines with TFF overexpression, we hypothesize that the overexpression of TFF3 is involved in prostate carcinogenesis via blocking mitochondria-mediated apoptosis.

Prostate tissues normally show low-level TFF3 expression^32^, suggesting that the overexpression (over 3,500 times) of TFF3 in PCa cells may have a role related to tumorigenesis. Previous studies have reported that the expression of TFF3 is relatively high in gastrointestinal epithelial cells and that it may induce tumorigenesis^17–20^, which supports the potential oncogenic role of TFF3 overexpression in the prostate. Through immunocytochemistry analysis, we found that the overexpressed TFF3 was located predominantly in the cytoplasm of PCa cells, which was consistent with a previous report suggesting that TFF3 is a biomarker of metastatic breast cancer^33^. When we silenced TFF3, both PCa cells showed significantly repressed growth and migration. These results suggest that TFF3 is involved in the migration and metastasis, in addition to the proliferation, of PCa cells. The repressed migration at an early time point (6–12 h) suggests that this result may not be affected by growth inhibition. Consistent with our results, previous studies have reported the association of TFF3 with tumor cell proliferation, migration, and metastasis in diverse solid tumors^22,28,34–38^. Regarding the mechanism of migration, our results suggest that TFF3 may induce migration through some chemokines and chemokine receptors but that MMP2 and MMP9 may not be involved in TFF3-induced migration.

**Fig. 7:**
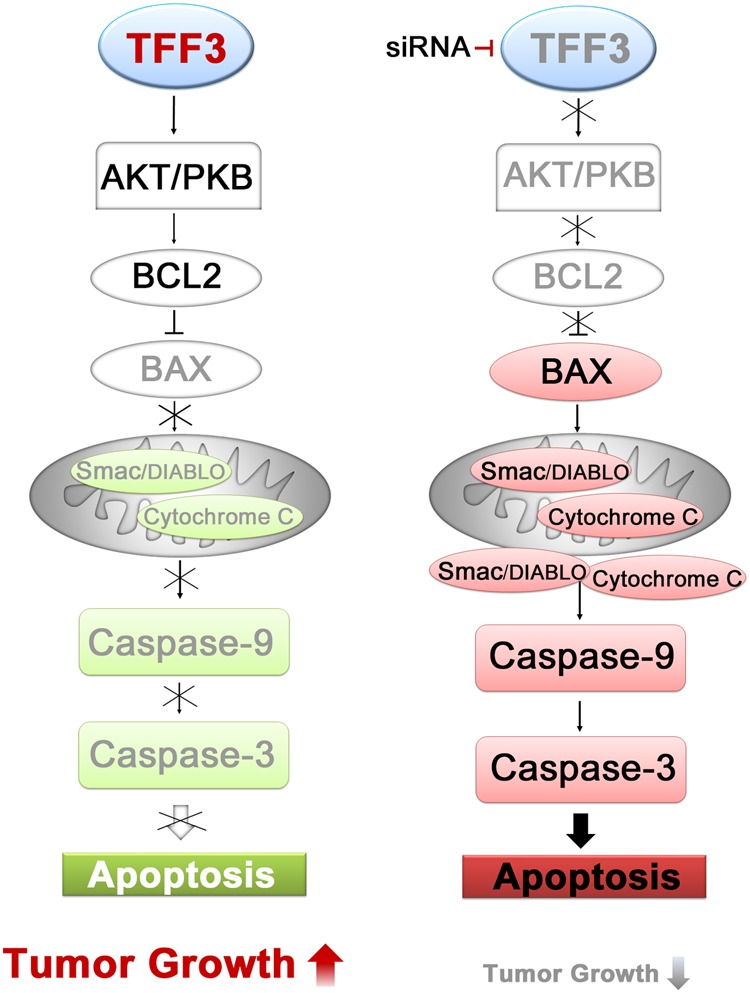
Schematic summary of the potential TFF3-associated molecular mechanism for prostate carcinogenesis. Overexpressed TFF3 activates the PI3K/AKT pathway, which blocks the mitochondria-induced apoptotic pathway and eventually induces prostate tumorigenesis. TFF3 silencing inhibits the phosphorylation of AKT-1 and the expression of BCL2, which increases the expression and mitochondrial translocation of BAX, and subsequently, the expression of proapoptotic proteins (cytochrome C and Smac/DIABLO) in the mitochondria and their release into the cytosol are elevated. In the cytosol, the elevated proapoptotic proteins activate caspase-9 and caspase-3 and induce apoptotic cell death

However, the downstream mechanism after PI3K/AKT activation is not well studied, especially in PCa. In this study, we focused on the downstream mechanism. As a downstream activator of the AKT signaling pathway, BCL2 increases cell survival by inhibiting the proapoptotic protein BAX^39^. As expected, TFF3 silencing decreased BCL2 expression and increased BAX expression^40–42^. We also confirmed the mitochondrial translocation of BAX, which is an essential event in mitochondrial apoptosis^39^. During apoptosis, mitochondrial proapoptotic proteins, such as cytochrome C, Smac/DIABLO and HtrA2/Omi, are known to be released into the cytosol and eventually activate caspase cascades via facilitating the formation of the apoptosome or blocking the antiapoptotic activity of apoptosis inhibitor protein family members^43–45^. Accordingly, the expression of the mitochondrial proapoptotic proteins cytochrome C and Smac/DIABLO was elevated after TFF3 silencing. Through the subcellular fractionation analysis, we also confirmed that these proteins were released from the mitochondria to the cytosol. However, we failed to check HtrA2/Omi in this study (data not shown). When we assessed caspase-3 and caspase-9, which are known downstream players in mitochondrial apoptosis, we found that the levels of cleaved caspase-3 and caspase-9 were elevated in TFF3-silenced cells. The level of Annexin V expression was also significantly elevated in TFF3-silenced cells. These results indicate that the repression of overexpressed TFF3 induces apoptosis via activating the signaling pathway related to effector caspases, such as caspase-3 and caspase-9, rather than via receptor-mediated cell death. Casado et al. demonstrated that colorectal cancer cells overexpressing TFF3 showed reduced sensitivity to chemotherapy by being resistant to apoptosis^46^. Hanisch et al. reported that TFF3 overexpression enhanced resistance to TNF-α/interferon gamma-induced apoptosis^47^. In the study by Perera et al. TFF3 overexpression was associated with reduced sensitivity to ionizing radiation in PCa cells^28^. When we checked cell cycle progression after TFF3 knockdown, there was no significant difference between TFF3-silenced and control cells (data not shown), suggesting that TFF3 silencing does not affect cell cycle progression in PCa. To verify the possibility of receptor-mediated apoptosis, we checked the expression of caspase-8, which is known to play a key role in receptor-mediated cell death signaling, and of three death receptors (TNFR1, FAS, and DR4), TNF-alpha and FADD. None of these showed a meaningful difference between TFF3-silenced and control cells. All the results support that TFF3 silencing induces the downstream signaling pathway of mitochondria-mediated apoptosis. Taken together, the data support the hypothesis that the TFF3-mediated antiapoptotic pathway plays a role in prostate carcinogenesis (Fig. 7).

There are several limitations of this study. First, regarding the TFF3-induced antiapoptosis pathway, we did not explore the whole potential pathway but rather focused on the downstream PI3K/AKT pathway, because activation of this pathway by TFF3 has been well studied^42,48–50^. Second, we did not perform TFF3 overexpression experiments. Instead, we observed 108 primary PCa specimens and found that TFF3 is significantly overexpressed in PCa. Third, we did not examine the role of TFF3 in PCa progression in an animal model. Furthermore, an in vivo study will be helpful in determining its oncogenic role. Fourth, although the expression of cytochrome C and Smac/DIABLO was clearly elevated, and they were released from the mitochondria to the cytosol after TFF3 silencing, we did not observe the expression of HtrA2/Omi in this study.

In summary, through the IHC analysis of 108 Korean PCa cases, we confirmed that TFF3 is significantly overexpressed in PCa. We also demonstrated that TFF3 is involved in the migration and metastasis, in addition to the proliferation, of PCa cells. We provide evidence that TFF3 overexpression is involved in antiapoptosis signaling in prostate tumorigenesis. This study provides a better understanding of the mechanism of prostate tumorigenesis and suggests TFF3 as a potential biomarker and therapeutic target of PCa.

## Electronic supplementary material

Supplementary Materials
