# Supplementary material for: Overexpression of TFF3 is involved in prostate carcinogenesis via blocking mitochondria-mediated apoptosis
Source: Exp Mol Med. 2018 Aug 23;50(8):110. doi: 10.1038/s12276-018-0137-7 (PMC6107499; doi:10.1038/s12276-018-0137-7)
Supplement: Supplementary file 1 — Supplementary Materials [file 12276_2018_137_MOESM1_ESM.pdf]

Supplementary Table 1. Sequence information of three TFF3-specific siRNAs.

	Sense strand sequence (5'→3')
siTFF3-1	UCAUGGCUGCCAGAGCGCUCUGCAU
siTFF3-2	CCUCCUGGACCAUGAAGCGAGUCCU
siTFF3-3	AGGAUCCCUGGAGUGCCUUGGUGUU

Supplementary Table 2. Clinicopathologic characteristics of the PCa in TMA.

	Cases
**Age at prostatectomy**	
Median (range)	63 (43-77)
**PSA at diagnosis**	
Median (range)	11.7 (0.1-135.5)
**Gleason score***	
5-6	10 (9.4 %)
7	43 (40.6 %)
8-9	53 (50.0 %)
**Pathological stage***	
T2-T3a	30 (75.0 %)
T3b and T4a	10 (25.0 %)
**Seminal vesical invasion***	
No	46 (80.7 %)
Yes	11 (19.3 %)

Our PCA TMA contains 108 PCa and 106 matched normal prostate tissues
* Cases with unknown status are not included.

Supplementary Table 3. Summary of TFF3 expression status determined by IHC analysis.

		IS	PCA (n=108)	Normal (n=106)	*p* value
Negative			49 (45.4%)	103 (97.2%)	$7.55X10^{-7}$
		0	27	89	
		1	10	6	
		2	5	4	
		3	7	4	
Positive			59 (54.6%)	3 (2.8%)	0.0058
	1+	4	2	1	
		5	4	0	
			6 (5.6%)	1 (0.9%)	
	2+	6	7	2	
		7	4	0	
		8	8	0	
		9	34	0	
			53 (49.1%)	2 (1.9%)	0.0062
Mean IS score			4.702	0.311	$2.52X10^{-24}$

IS, Immunohistochemistry score.

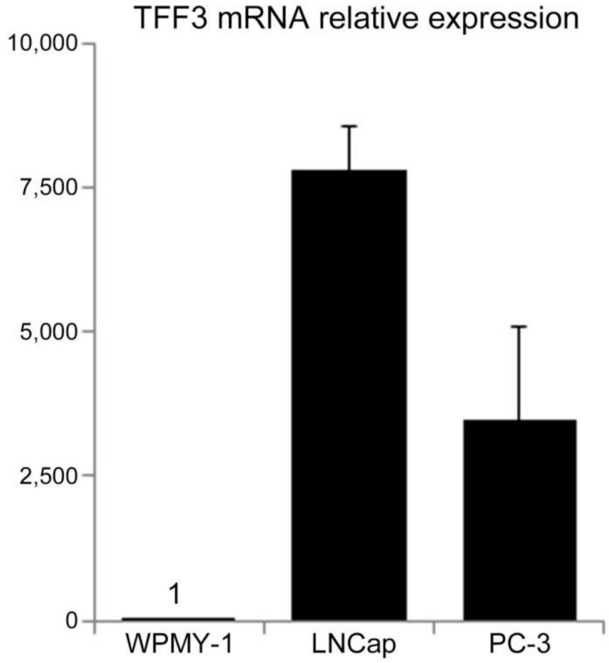

Supplementary Figure 1. Relative expression levels of TFF3 in the Pca cell lines (LNCap and PC-3) compared with normal prostate cell line (WPMY-1). The levels of TFF3 mRNA expression in LNCap and PC-3 were highly up-regulated than in normal prostate cells. Y axis represents relative values of TFF3 mRNA detected by real-time qRT-PCR. Relative expression values were presented as mean ±SEM of three independent measurements. The TFF3 mRNA level in WPMY-1 was assigned as value of 1. The relative TFF3 mRNA expression of cell lines was standardized to glyceraladegyde-3-phosphate dehydrogenase (GAPDH).

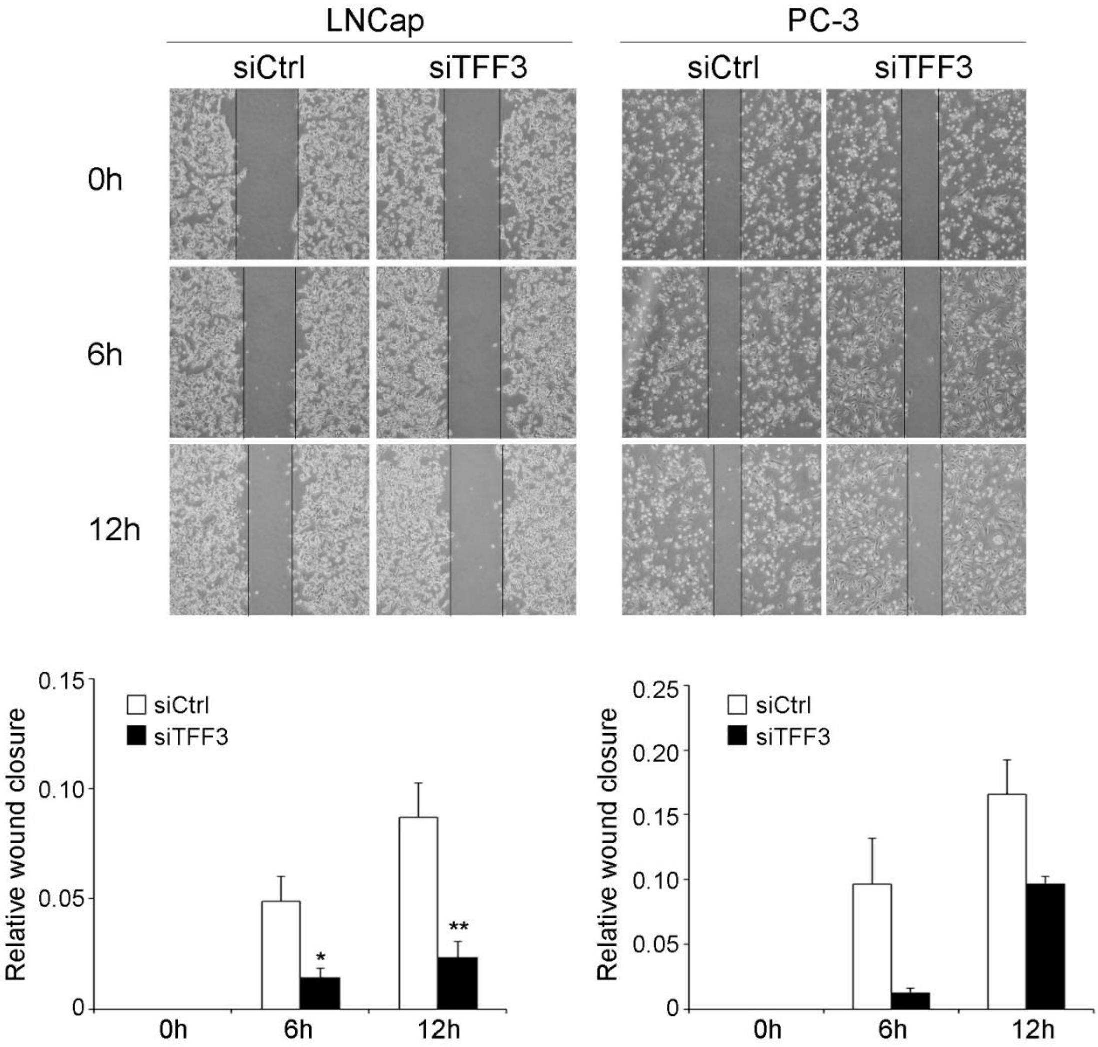

Supplementary Figure 2. Effect of TFF3 silencing on PCa cell motility. The images show cell migrations monitored by scratch wound healing assay at different time points (0, 6h, and 12h) after transfection. Bar charts at the right side of the images represent the relative wound closure. Relative wound closure was calculated using the formula: wounded area invaded by cells/wounded area at 0 hour. Relative wound closure value was mean ± SEM of the results from three independent experiments. *$P < 0.05$; **$P < 0.01$.

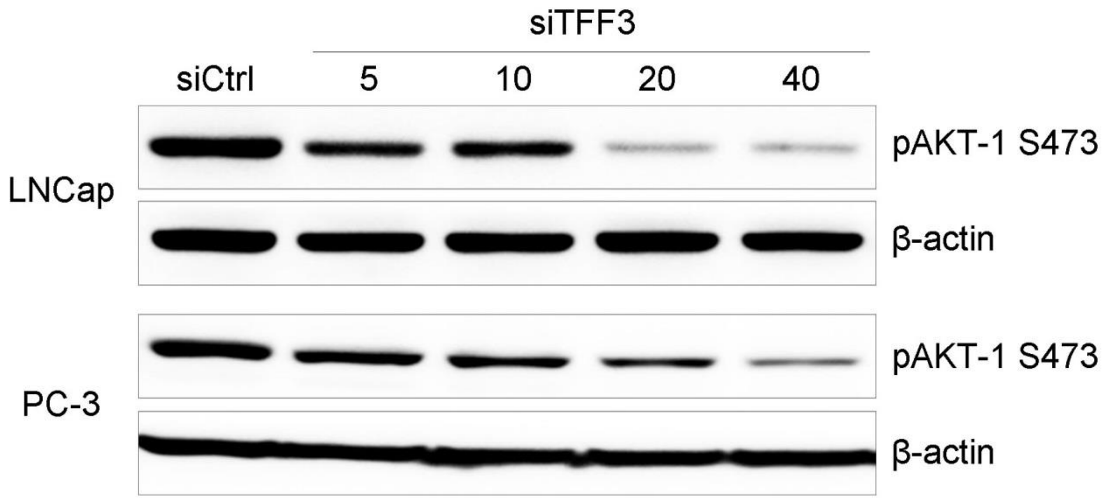

Supplementary Figure 3. Expression levels of phosphorylated AKT-1 after treatment of siTFF3 (5, 10, 20 and 40 nM) in PCa cells. siTFF3 treated PCa cells showed reduced levels of phosphorylated AKT-1 compared with siCtrl treated cells siTFF3 dose dependently.

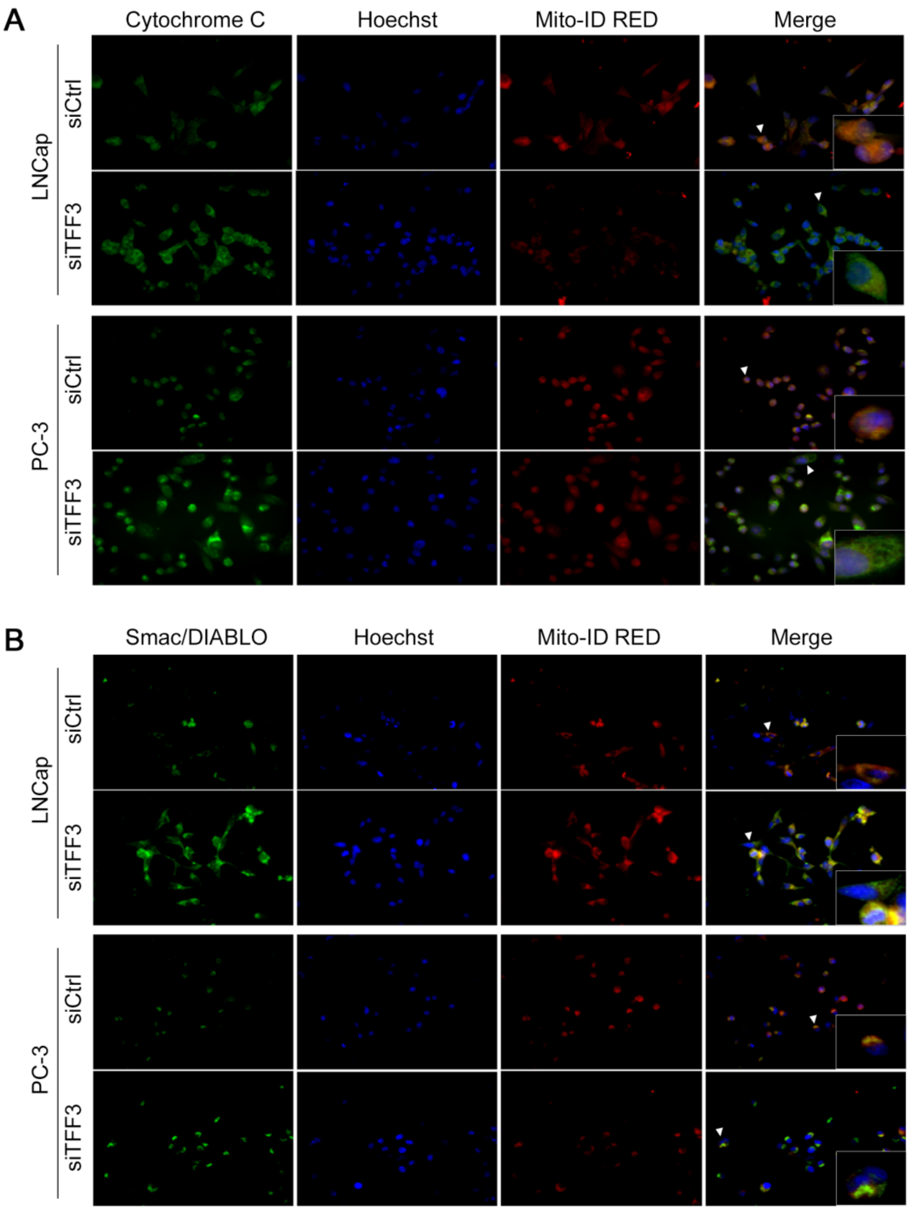

Supplementary Figure 4. Expression of mitochondrial pro-apoptotic proteins after TFF3 silencing. (A, B) Expression and subcellular localization of cytochrome C and Smac/DIABLO (both green colors) (400X). Mitochondria (red: Mito-ID RED) and nuclei (blue: Hoechst 33342) are also shown (400X). Immunocytochemistry showed that the expression levels of Cytochrome C and Smac/DIABLO were elevated and they were released from mitochondria to cytosol in both TFF3-silenced cells. Arrowheads indicate the cells of each inset of LNCap and PC-3 cells transfected with siTFF3 and siCtrl.

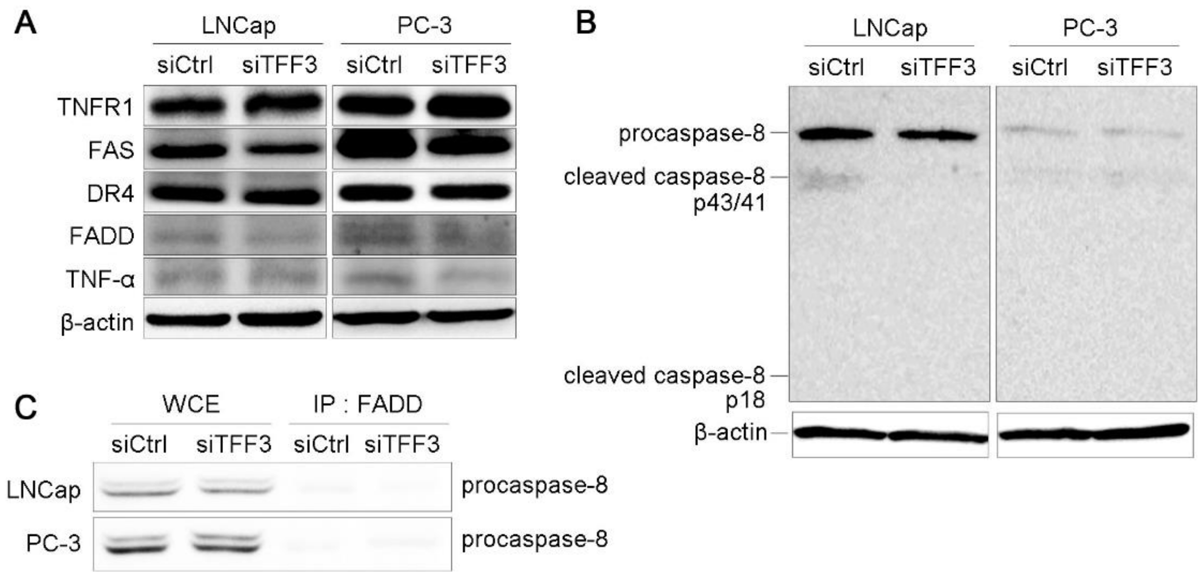

Supplementary Figure 5. TFF3-silencing does not stimulate death receptor-mediated (extrinsic) apoptotic pathway. (A) Expression levels of death receptors (TNFR1, FAS and DR4), Fas-associated protein with death domain (FADD) and TNF alpha after TFF3-silencing in PCa cells. The death receptors-related factors did not show significant difference between TFF3-silenced and control cells. (B) Expression levels of caspase-8 after TFF3-silencing in PCa cells (LNCap and PC-3). Both of 57 kDa-sized full length caspase-8 and 43 kDa-sized cleaved caspase-8 did not show significant difference between TFF3-silenced and control cells. (C) TFF3-silencing did not promote the formation of FADD-caspase-8 complex. Immunoprecipitation (IP) with an anti-FADD Ab was followed by immunoblot detection of procaspase-8. WCE, whole-cell extract.

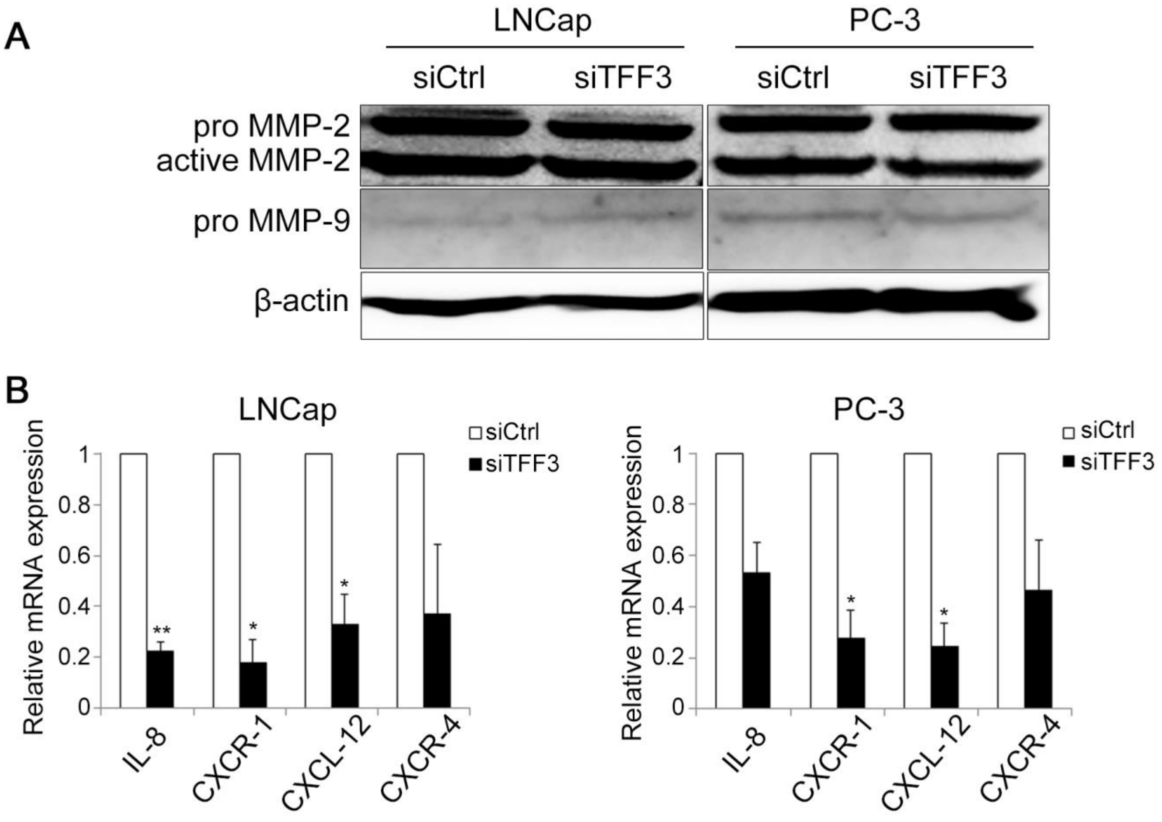

Supplementary Figure 6. Expression of migration-related molecules (MMP2, MMP9, CXCR1, CXCR4, CXCL12 and IL8) after TFF3-silencing in PCa cells. (A) Expression of MMP2 and MMP9 was observed by western bolt analysis. (B) Expression of the other molecules was observed by qRT-PCR. Expression levels of MMP2 and MMP9 was not different between TFF3-silenced and control cells but expressions of CXCR1, CXCR4, CXCL12 and IL8 was downregulated in TFF3-silenced cells. *$P < 0.05$; **$P < 0.01$
